# Antierythropoietin Antibody Production Is Not Associated with Malaria and Malaria-Related Anaemia in Humans

**DOI:** 10.1155/2019/5398732

**Published:** 2019-02-14

**Authors:** Otchere Addai-Mensah, Daniel Gyamfi, Francis Agyei Amponsah, Max Efui Annani-Akollor, Kwabena Owusu Danquah, Lillian Boateng, Eddie-Williams Owiredu, Edward Y. Afriyie, Richard Vikpebah Duneeh, Renate Asare, David Ofosu Ntiamoah, Richard Boateng

**Affiliations:** ^1^Department of Medical Laboratory Technology, Faculty of Allied Health Sciences, Kwame Nkrumah University of Science and Technology, Kumasi, Ghana; ^2^St. John of God Hospital, Duayaw Nkwanta, Sunyani, Ghana; ^3^Department of Molecular Medicine, School of Medical Sciences, Kwame Nkrumah University of Science and Technology, Kumasi, Ghana; ^4^Department of Haematology, Komfo Anokye Teaching Hospital, Kumasi, Ghana; ^5^Department of Serology, Komfo Anokye Teaching Hospital, Kumasi, Ghana

## Abstract

**Introduction:**

The pathophysiology of malaria-related anaemia is not fully understood although increased destruction of parasitized and nonparasitized erythrocytes, as well as inadequate erythropoiesis, has been proposed. Circulating antierythropoietin (anti-EPO) antibodies have also been implicated in malaria and malaria-related anaemia in mice. However, studies on this association have not been investigated in humans. This study therefore determined the prevalence of anti-EPO antibody production and assessed its association with malaria and malaria-related anaemia in humans.

**Methods:**

A total of 86 children aged 1-10 years (57 children with malaria serving as the case group and 29 healthy children serving as control), all residents of Duayaw Nkwanta, Ghana, were recruited for this case-control study. Venous blood was collected for thick and thin films for malaria microscopy, full blood count by automated haematology analyzer, and antierythropoietin antibody and erythropoietin estimation by sandwich ELISA method.

**Results:**

Out of the 86 participants recruited, only 3 (3.5%) were positive for anti-EPO antibody; 2.3% of the case group; and 1.2% of the control group. There was no association between the cases and the controls in the production of anti-EPO antibodies. Erythropoietin concentration was significantly higher in malaria-related anaemic subjects (p=0.032).

**Conclusion:**

Antierythropoietin antibodies are not associated with malaria infection and malaria-related anaemia in humans. Erythropoietin concentration is associated with malaria-related anaemia.

## 1. Introduction

Malaria continues to exact an enormous toll, claiming the life of a child under five years every two minutes in Sub-Saharan Africa [1]. Five species of Plasmodium (*falciparum, malariae, ovale, vivax, *and* knowlesi*) are known to cause human malaria with* P*.* falciparum *being the major cause of the disease with debilitating symptoms and complications such as severe malaria-related anaemia in Africa, accounting for 86% of all infections [2–4].

During the initial stages of malaria infection, there is haemoglobin digestion by* Plasmodia *sp. resulting in anaemia which significantly correlates with the degree of parasitaemia [5], though studies have shown the occurrence of severe malarial anaemia at low parasitaemia in the nonimmune individual [6, 7].

Although the pathogenesis of malaria-related anaemia is not fully understood, studies have shown that it is multifactorial, consisting of increased destruction of parasitized and nonparasitized erythrocytes as well as impaired regeneration of erythrocytes or inadequate erythropoiesis [5, 8, 9]. A study by Leowattana et al. showed that reticulocyte response is delayed in people with acute malaria infection, suggesting a transient suppression of the normal erythropoietin (EPO) response during periods of tissue hypoxia [10], leading to anaemia.

There are, however, conflicting reports on levels of EPO during episodes of malaria. Some studies observed normal to increased EPO levels in children with malaria-related anaemia [11, 12] while others showed that there are reduced EPO levels, according to the degree of anaemia [5].

In 2013, Helegbe et al. proposed the possible role of anti-EPO in malarial anaemia. They evaluated anti-EPO in relation to the pathogenesis of malaria-related anaemia in different strains of semi-immune mice infected with* Plasmodium berghei* and established that malaria infection was associated with anti-EPO antibody production in some strains of mice [13]. However, due to genetic diversity between mice and man as well as in the infecting* Plasmodium* species, findings in murine models may not hold in humans. This study therefore established the prevalence of anti-EPO antibodies and assessed its association with malaria and malaria-related anaemia among Ghanaian children.

## 2. Materials and Methods

### 2.1. Study Design/Setting

This case-control study was conducted from August to September 2017 at the St John of God Hospital (a municipal hospital) and the Prince of Peace Academy, both in Duayaw Nkwanta.

Duayaw Nkwanta is a town and the capital of Tano North District, a district in the Brong-Ahafo Region of Ghana with a settlement population of about 17,476 [14].

The Brong-Ahafo region has a perennial malaria transmission, with the predominant parasite being* P. falciparum*. Brong-Ahafo region recorded 9,294 cases of malaria in 2011 and 7,660 in 2012. A cross-sectional survey conducted in 2006/2007 recorded the prevalence of malaria parasitaemia among children in Brong-Ahafo region to be 22.8% [15].

### 2.2. Participants' Recruitment

A simple randomized sampling technique was used to recruit fifty-seven (57) children, who were diagnosed as having malaria at the out-patient department (OPD) of St John of God Hospital, as the case group whiles twenty-nine (29) apparently healthy children served as the control group.

### 2.3. Inclusion and Exclusion Criteria

Children, between the ages of 1 and 10, whose parents' consented to the study, were included. Children with malaria with coexisting helminthic infection or sickling positivity were excluded from the study.

### 2.4. Ethical Approval

Ethical approval was obtained from the Committee on Human Research Publication and Ethics (CHRPE) of the School of Medical Sciences at Kwame Nkrumah University of Science and Technology and Komfo Anokye Teaching Hospital, Kumasi (CHRPE/AP/514/17). Written informed consent was obtained from all parents and/or guardians of participants.

### 2.5. Sample Collection and Laboratory Investigations

Ten (10) milliliters of blood was collected from the antecubital vein of each respondent. Five (5) milliliters was dispensed into K_3_ EDTA tubes whiles the remaining 5 ml was dispensed into gel separator tubes, placed in a centrifuge, and spun at 3000 rpm for 10 minutes to obtain the serum.

Whole blood in EDTA tubes was used for full blood count (FBC) and thick and thin films for malaria microscopy. The full blood count was performed using Sysmex KX-21N auto analyzer (Sysmex Corporation, Japan). The thick and thin blood films were 10% Giemsa-stained for malaria parasite identification, count, and density. Parasites observed were all* Plasmodium falciparum* based on the morphological characteristics. Malaria was defined by the presence of malaria parasite in blood film. Blood films were declared negative if no parasites were seen in 200 oil-immersion fields as described by Squire et al. [16]. Malaria parasite density was calculated based on the formula:

(Number of parasites counted/WBC counted) × WBC count/*μ*l of participant.

Malaria-related anaemia was defined as haemoglobin level ≤ 11.0g/dl [17].

Serum obtained was used for EPO and anti-EPO antibody estimation using Human EPO Sandwich ELISA kit and Human EPO Antibody Sandwich ELISA kit (Sunlong Biotech Co., China) respectively.

### 2.6. Statistical Analysis

Categorical variables were presented as frequencies (percentages) and test for association was performed using the Chi-square test statistic. Parametric continuous variables were expressed as the mean ± standard deviation and nonparametric variables as median (interquartile range). Independent t-test and Mann-Whitney U test were used to test for significance of differences between parametric and nonparametric variables, respectively. Binary logistic regression was used to ascertain the odds of the participants with malaria to produce anti-EPO antibodies. A p-value < 0.05 was considered significant. All data analysis was performed using the IBM SPSS 20.0.

## 3. Results

The demographic, clinical, and haematological characteristics of studied population are shown in Table 1. Out of the 86 children enrolled, 66.3% had malaria (confirmed by microscopy), constituting the case group, and 29 (23.7%) were healthy, serving as controls. The mean age of the entire study population was 5.3 ± 2.6 years with majority of them (both the case and the control participants) being males.

The case group had statistically significant higher temperature (37.9 ± 0.97°C vs 36.9 ± 0.12°C, p<0.0001) and total WBC (9.4 ± 3.71 x 10^9^/L vs 7.6 ± 1.60 x 10^9^/L, p=0.012) compared to the control group and a statistically significant lower haemoglobin level (10.4± 1.95 g/dL vs 13.1± 0.85 g/dL, p<0.0001), MCH [24.9±1.99 pg vs 30.0±2.46 pg, p<0.0001], and platelet count [161.0 x 10^9^/L (108.5- 223) vs 458 .0 x 10^9^/L (388- 510), p<0.0001] (Table 1).

Similarly, EPO concentration was lower in the case group [57.49(47.65-89.86) pg/ml] compared to the control group [71.86(62.41-88.20) pg/ml] though the difference was not statistically significant (p=0.264) (Table 1).

Table 2 shows the association between anti-EPO antibody and malaria among the study participants. Out of the 86 participants recruited, only 3 (3.5%) were positive for anti-EPO antibody, consisting of 2.3% of the case group and 1.2% of the control group. There was no association between the cases and the controls in the production of anti-EPO antibodies (Table 2).

Haematological and immunological parameters among participants with malaria stratified by anaemic status are shown in Table 3. More than half (59.6%) of the participants with malaria were found to be anaemic, with the anaemia been more prevalent among younger participants (3.74 ± 2.09 years' vs 6.04± 2.25 years; p<0.0001) (Table 3). EPO concentration was significantly higher in malaria-related anaemic subjects (p=0.032) (Table 3). Though not statistically significant, the level of parasitaemia was higher in the malaria-related anaemic children compared to the nonanaemic children (p=0.222). There was however no association between anti-EPO antibodies and malaria-related anaemia (Table 3).

## 4. Discussion

Anti-EPO antibodies have been implicated in the anaemia associated with many diseases including HIV-1/AIDS, systemic lupus erythematosus, and pure Red Cell Aplasia among others [18–20].

Recent studies have also shown an association between antierythropoietin antibodies and malaria-related anaemia in murine models. A study by Tsubata et al. found that treating infected mice with exogenous antierythropoietin (anti-EPO) antibodies provides protection against malaria infection [21]. Another study by Helegbe et al. observed the role of anti-EPO antibodies in malarial anaemia by evaluating anti-EPO antibody in relation to the pathogenesis of malaria-related anaemia in different strains of semi-immune mice infected with* Plasmodium berghei*, where they established that malaria infection was associated with anti-EPO antibody production in some strains of mice [13]. These suggest that anti-EPO antibodies may play a significant role in malaria and malaria-related anaemia. However, due to genetic diversity between different species, findings in mice may not hold in humans. This study therefore established the prevalence of anti-EPO antibodies and assessed its association with malaria and malaria-related anaemia among Ghanaian children.

This study observed the expected high temperature among participants with malaria compared to the controls (Table 1), consistent with previous studies [22–24]. The mechanism of fever, as associated with malaria infection, is not well understood; however, a recent study in mice suggests that malaria-related fever is caused by stimulation of TLR9 by malarial DNA through hemozoin, an insoluble haemoglobin digestion product [25]. Participants with malaria also presented with significantly elevated WBC compared to the controls (Table 1), consistent with a study by Ladhani et al. [26].

Various mechanisms of malarial anaemia have been investigated including increased RBC destruction through rupture of parasitized red blood cells (PRBCs) [27], phagocytosis of PRBCs [28], hypersplenism [29], and hapten-induced intravascular haemolysis [30] as well as decreased RBC production through inflammation-induced erythroid hypoplasia [31], imbalance of cytokines [32]m and suppression of erythropoietin synthesis [33]. In this study, a significantly lower haemoglobin level and MCH was also observed among subjects with malaria compared to controls, indicating anaemia among participants with malaria (Table 1). This finding is consistent with previous studies in Ghana [34, 35].

Furthermore, as an obligate intracellular parasite,* Plasmodia *sp. do not affect only red blood cells but also platelets as these cells originate from a common stem cell [36]. A significantly lower platelet count was also observed among subjects with malaria compared to controls in this study (Table 1), attributable to splenic sequestration of platelet in malaria. This finding is consistent with previous studies by Jadhav et al. [37], Patel et al. [38], and Shaikh et al. [39]. Additionally, though not statistically significant, it is worth noting that EPO concentration was lower among subjects with malaria compared to the controls (Table 1). Earlier reports show that TNF-*α* is elevated in malaria due to inflammation [40, 41]. On the other hand, TNF-*α* has also been reported to inhibit EPO production and EPO-induced erythroid progenitor cell proliferation [42]. This could be a possible reason for the reduced level of EPO among children with malaria compared to those without malaria. However, due to the fact that we did not assess the level of TNF- *α* in this study, we could not conclude about the impact of TNF- *α* on EPO concentration; thus this association requires further studies.

The prevalence of anti-EPO antibodies production among the entire study population was 3.5%; 2.3% in the case group; and 1.2% in the control group (Table 2). Chi-square test for independence showed no significant association between malaria and production of anti-EPO antibodies (Table 2), contradicting previous studies in mice [13, 21]. Moreover, there was no association between anti-EPO antibodies and malaria-related anaemia (Table 3), indicating that the presence of the antibody does not predispose to malaria-related anaemia. This disparity could be attributed to the different species studied as well as the different infecting species of Plasmodium.* Plasmodium berghi Anka* was the malaria parasite examined in the study by Helegbe et al. in mice; however, the predominant Plasmodium species responsible for malaria infection in humans in Ghana is* Plasmodium falciparum*.

Additionally, during falciparum malaria infection, there is loss of both parasitized and nonparasitized erythrocytes through parasite maturation and antibody sensitization, respectively, leading to anaemia. Erythropoietin (EPO), on the other hand, is crucial to sustain the proliferation and differentiation of erythroid cells. EPO is bound by circulating erythrocytes and studies have shown that reduction in circulating RBCs results in a relatively high concentration of unbound EPO which in turn stimulates erythrocyte production in the bone marrow [43, 44]. In this study, majority of the participants with malaria were found to be anaemic (59.6%); and EPO concentration was significantly higher in malaria-related anaemic subjects compared to their nonanaemic counterparts (Table 3). This finding is consistent with a study by Verhoef et al. among Kenyan children [12], who reported that children with malaria had both reduced haemoglobin and increased concentrations of erythropoietin. An earlier study by Srichaikul et al. [45] and Kurtzhals et al. [46] also reported inadequate erythroid response despite increased concentrations of erythropoietin in malaria. The increased production of EPO may be attributable to the response of EPO to anaemia or hypoxia. However, the finding suggests that the increased production of EPO is unable to resolve the anaemia, possibly due to the initiation of apoptosis of nascent erythroid cells [47].

This study is however limited by the small sample size used which may have encumbered the full exploration of the association between anti-EPO and malaria. Future studies may focus on a larger sample size.

## 5. Conclusion

Antierythropoietin antibodies are not associated with malaria infection and malaria-related anaemia in humans. Erythropoietin concentration is associated with malaria-related anaemia.

## Figures and Tables

**Table 1:** Demographic, clinical, and haematological characteristics of studied population.

Variables	Total; N=86	Case; 57 (66.3%)	Control; 29 (33.7%)	P-values
Age (years)	5.3±2.6	4.67± 2.4	6.5± 2.4	**0.001‡**
Sex				0.473*∗*
Male	50(58.1)	32(37.6)	18(21.2)	
Female	36(41.9)	25(29.4)	10(11.8)	
Temperature (°C)	37.7±0.93	37.9 ± 0.97	36.9± 0.12	**<0.0001‡**
RBC (x10^6^/*µ*L)	4.3±0.73	4.2±0.8	4.4±0.46	0.135‡
Haemoglobin level (g/dL)	11.3±2.10	10.4± 1.95	13.1± 0.85	**<0.0001‡**
MCV (fL)	75.9±11.0	75.7±9.15	76.5±14.31	0.755‡
MCH (pg)	25.9±3.2	24.9±1.99	30.0±2.46	**<0.0001‡**
WBC (x10^3^/*µ*L)	8.8± 3.27	9.4± 3.71	7.6± 1.60	**0.012‡**
Platelet count (x10^3^/*µ*L)	223 (145- 413)	161.0 (108.5- 223)	458 .0 (388- 510)	**<0.0001†**
EPO concentration (pg/ml)	65.36 (53.40- 89.26)	57.49 (47.65- 89.86)	71.86 (62.41- 88.20)	0.264†

‡Independent t-test and †Mann-Whitney U test were used to test for significance of differences between parametric and nonparametric variables, respectively. *∗*Chi squared test was used to test for association between categorical variables. A p-value < 0.05 was considered significant (*p* values of significant variables are in bold print).

**Table 2:** Association between anti-EPO antibody and malaria among the study participants.

Anti-EPO antibody	Total	Negative	Positive	p-value	OR (95% CI)	p-values
Controls	29(33.7)	28(32.6)	1(1.2)	1.000†	1(referent)	
Cases	57(66.3)	55(63.9)	2(2.3)		0.982(0.085-11.312)	0.988

†Fisher exact test was performed to test for association between categorical variables. Binary logistic regression analysis was used to evaluate the odds of malaria to induce anti-EPO production (presented as odds ratio with 95% confidence interval). *p* < 0.05 was considered statistically significant.

**Table 3:** Haematological and immunological parameters among participants with malaria stratified by anaemic status.

Variables	Non-anaemic; 23(40.4%)	Anaemic; 34(59.6% )	p- value
Mean Age ±SD (years)	6.04 ± 2.25	3.74 ± 2.09	**<0.0001**†
Parasite density (par/uL of blood)	45142 (4320- 116919)	78394 (16168- 179234)	0.222‡
EPO concentration (pg/ml)	52.65 (37.52- 73.52)	70.95 (53.18-101.66)	**0.032**‡
Anti- EPO antibodies			1.000**∗**
Positive	1(1.8)	1(1.8)	
Negative	22(38.6)	33(57.8)	

*∗*Fisher exact test was performed to test for association between categorical variables. †Independent t-test and ‡Mann-Whitney U test were used to test for significance of differences between parametric and nonparametric variables, respectively. A p-value < 0.05 was considered significant (*p* values of significant variables are in bold print).

## Data Availability

All relevant data are within the article.
